# Blood Testis Barrier and Somatic Cells Impairment in a Series of 35 Adult Klinefelter Syndrome Patients

**DOI:** 10.3390/ijms20225717

**Published:** 2019-11-14

**Authors:** Maria Grazia Giudice, Maxime Vermeulen, Christine Wyns

**Affiliations:** 1 Department of Gynecology-Andrology, Cliniques Universitaires Saint-Luc, 1200 Brussels, Belgium; maria.giudice@uclouvain.be; 2 Gynecology-Andrology Research Unit, Institut de Recherche Expérimentale et Clinique, Medical School, Université Catholique de Louvain, 1200 Brussels, Belgium; m.vermeulen@uclouvain.be

**Keywords:** Klinefelter syndrome, fertility preservation, blood–testis barrier, Sertoli cells, Leydig cells

## Abstract

Klinefelter Syndrome (KS) is the most common genetic cause of infertility in men. Degeneration of the testicular tissue starts in utero and accelerates at puberty with hyalinisation of seminiferous tubules, spermatogonia apoptosis and germ cell maturation arrest. Therefore, fertility preservation in young KS boys has been proposed, although this measure is still debated due to insufficient knowledge of the pathophysiology of the disease. To better understand the underlying mechanisms of testicular failure and germ cell loss, we analysed functional and morphological alterations in the somatic compartment of KS testis, i.e., Sertoli cells, including the blood–testis barrier (BTB) and Leydig cells (LC). We compared three populations: 35 KS 47,XXY non-mosaic patients, 28 Sertoli-cell-only (SCO) syndrome patients and 9 patients with normal spermatogenesis. In KS patients the expression of BTB proteins connexin-43 and claudin-11 assessed with a semi-quantitative scoring system appeared significantly reduced with a disorganised pattern. A significant reduction in seminiferous tubules expressing androgen receptors (AR) was observed in KS compared to normal spermatogenesis controls. INSL3 expression, a marker of LC maturation, was also significantly reduced in KS compared to patients with normal spermatogenesis or SCO. Hence, the somatic compartment impairment in KS could be involved in degeneration of seminiferous tubules.

## 1. Introduction

Klinefelter Syndrome (KS) is the most frequent sexual chromosomal aneuploidy in men, characterised by the presence of an extra “X” chromosome. It occurs in 1/600 newborns [1]. KS is the main genetic cause of male infertility, due to loss of germ cells (GCs) including the spermatogonial stem cells (SSCs), as well as causing tubular atrophy, GC maturation arrest, fibrosis and hyalinisation of the testicular tissue associated with Leydig cell (LC) hyperplasia [2]. As testicular tissue degeneration has already begun in the embryo stage and accelerates during puberty [3], there is a huge debate on the relevance of proposing fertility preservation options before puberty, or during the pubertal transition period, in KS boys [4,5,6,7,8]. Indeed, almost 40% of patients have some remaining foci where mature sperm may be recovered by testicular sperm extraction (TESE) during early adulthood and so far knowledge on molecular mechanisms involved in degeneration of seminiferous tubules (STs) and on the reproductive potential of spermatogonia in KS is very limited and uncertain (for review see Giudice et al. [9]).

In KS patients, a number of alterations involving Sertoli cells (SCs), which play a central role in both testicular development and germ cell differentiation, have been reported [10]. Indeed, while physiologically the release of anti-Müllerian hormone (AMH) by SCs decreases during puberty and is arrested when SCs achieve maturation, in KS a delay in disappearance of AMH expression during puberty and a decline of circulating AMH in adulthood have been described, probably as a result of disrupted regulatory mechanisms in the pituitary–gonadal axis [11,12]. A reduced SC expression of nuclear androgen receptors (AR) whose signalling is essential for spermatogenesis [13] was also shown in KS boys [12]. 

The blood–testis barrier (BTB) has been poorly studied in KS. This dynamic structure take place during puberty [14] and is composed by different junction types such as tight junctions including claudin-11 (CLD11) to assure a polarity between basal and apical seminiferous tubule compartments [15], gap junction, such as connexin 43 (CNX43) that allow communication between two SCs and permit the passage of small molecules between adjacent cells [16], and adhesion and adapter proteins like zonula occludens-1 (ZO1) necessary to fix proteins to the SC’s membrane [17]. As it is essential to protect haploid GCs from the innate immune system and to prevent the intercellular diffusion of molecules [18], any impairment of the BTB might cause testicular microenvironment deregulation and spermatogenesis failure [19]. The possibility that it may be affected in KS has been raised and based on one case report it was suggested that CNX43 could be missing [20].

The syndrome is further characterised by LCs hyperplasia where LCs are grouped in clusters [21] and show, after an initial rise of insulin-like peptide 3 (INSL3) secretion at onset of puberty, an abnormal release pattern with a tendency of levelling off despite increased LH (luteinizing hormone) levels [22]. Transcriptomic analyses also contributed to unravelling disease features. An over-expression of genes encoding the steroidogenic acute regulatory protein (StAR) mediating the passage of cholesterol inside the mitochondrial membrane, which is essential for testosterone biosynthesis in LCs [23], and of other enzymes related to the androgens production, have been reported in three KS patients [24].

As data from the literature are still too scarce to enable understanding of testicular tissue and cell impairment in KS, and as only case report and a small series of KS patients were reported, we further investigated the somatic testicular compartment in adult KS patients. We focused on SC and LC maturation and function, as well as on main BTB proteins, in a large cohort of KS patients compared to: (1) Sertoli-cell-only (SCO) patients with normal karyotype and anatomopathological features similar to KS, and (2) to a group of patients with normal spermatogenesis (NSP) and normal karyotype.

## 2. Results

### 2.1. Blood–Testis Barrier Protein Expression Patterns

**Figure 1:**
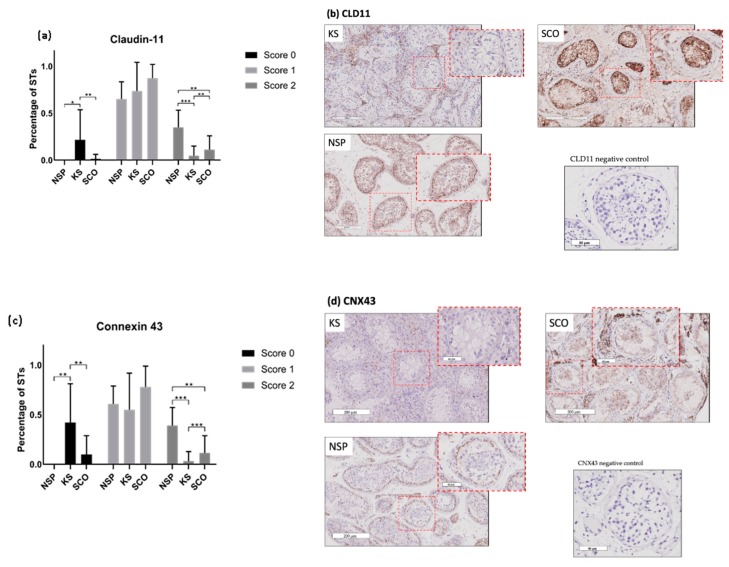
Expression of CLD11 (**a**–**b**) and CNX43 (**c**–**d**) according to the different scores (0, 1, 2) in the three different populations: normal spermatogenesis (NSP), Klinefelter Syndrome (KS) and Sertoli-cell-only (SCO). The ratios between the number of seminiferous tubules (STs) of each score and total number of STs per slide (expressed as a percentage) are presented: (**a**) shows reduction of CLD11 expression in KS compared to NSP * *p* ≤ 0.02 and to the SCO group ** *p* ≤ 0.003, and a significant reduction of organised pattern (score 2) in KS compared to NSP *** *p* ≤ 0.002 and to SCO ** *p* ≤ 0.006. (**b**–**d**) STs sections (scale bar = 200 μm; magnification ×40 in red dotted square) with score 0 in KS patients, score 2 in NSP, score 1 in SCO group for CLD11 (**b**), CNX43 (**d**) and negative controls (omission of primary antibody). (**c**) shows reduction of CNX43 expression in KS compared to NSP ** *p* ≤ 0.002 and to SCO ** *p* ≤ 0.001 and also a reduced organised pattern in KS compared to NPS *** *p* ≤ 0.0001 and to SCO *** *p* ≤ 0.003.

Expression patterns of CLD11 and CNX43 are shown in Figure 1. A total of 3639 STs with a mean of 28.5 ± 27.9 STs per patient were analysed. 

A mean of 22% ± 32% STs for CLD11 scored as 0 (absence of protein) in KS, which was significantly higher than in NSP (mean of 0% STs; *p* ≤ 0.02) or in SCO group (mean of 1% ± 5% STs; *p* ≤ 0.003). On the other hand, a mean of 5% ± 10% STs in KS presented a score 2 corresponding to a well-organised distribution of the protein, which was lower in KS than in NSP (mean of 35% ± 18% STs; *p* ≤ 0.002) and SCO (mean of 11% ± 15% STs; *p* ≤ 0.006) (Figure 1a,b). No significant differences were found between groups for % STs with score 1. 

A mean of 42 ± 39% STs were scored 0 (absence of expression) for the gap junction protein CNX43 in KS which was statistically higher than in NSP (mean of 0% STs; *p* ≤ 0.002) or SCO (mean of 10% ± 19% STs; *p* ≤ 0.001) groups. On the other hand, a mean of 3% ± 10% STs were scored 2 (well-organised protein distribution) corresponding to a reduction of the protein pattern organisation in KS compared to NSP (mean of 39% ± 18% STs; *p* ≤ 0.0001) and to SCO (mean of 12% ± 18% STs; *p* ≤ 0.003) (Figure 1c,d). No differences were found for score 1 STs percentages between the three groups. 

No STs presented a score 0 (absence of the protein staining) for ZO1 in all three groups. A mean of 68% ± 31% STs, 59% ± 28% STs and 74% ± 18% STs were scored as 1 (apical–basal distribution protein pattern) in KS, NSP and SCO patients, respectively, with no differences between groups. No differences were found for score 2 (basal-organised pattern) ZO1 STs percentages between groups showing a mean of 32% ± 31% STs in KS, 40% ± 28% STs in NSP and 26% ± 18% STs in SCO patients. 

### 2.2. Sertoli Cell Maturation and Function 

A total of 3136 STs with a mean of 25.8 ± 24.8 STs per patient were analysed. No significant difference was found when we compared the AMH expression in the three groups. In KS a mean of 67% ± 37% STs did not present the protein AMH (score 0), and a mean of 33% ± 37% STs presented a score 1. In NSP a mean of 71% ± 35% STs presented a score 0 and a mean of 29% ± 35% STs presented a score 1. In SCO group a mean of 59% ± 34% STs had a score 0 and a mean of 41% ± 34% STs had a score 1. Concerning GDNF expression (score 1), a mean of 99% ± 6% STs, 100% ± 0% STs and 100% ± 0% STs presented the protein expression (score 1) in KS, NSP and SCO, respectively. 

**Figure 2:**
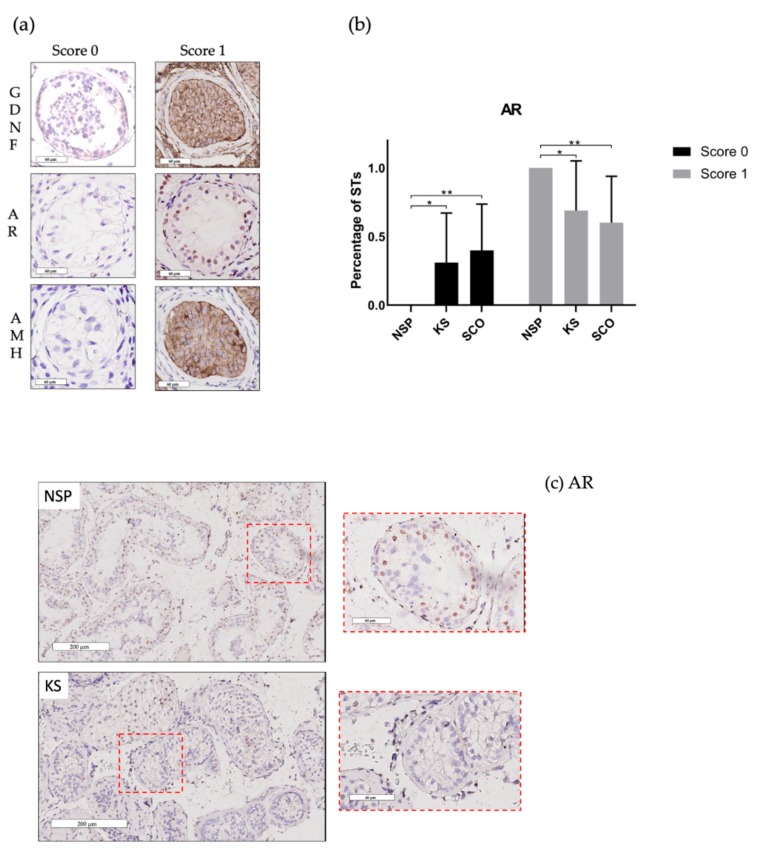
SCs protein expressions. (**a**) Two different scores were attributed to each ST with a preserved basement membrane: 0, absence of staining and; 1, presence of staining localised in the cytoplasm for GDNF and AMH; nuclear staining for androgen receptor (AR) (scale bar = 60 μm). (**b**) AR in KS are significantly reduced compared to the NSP group * *p* ≤ 0.01. AR are reduced in SCO patients compared to NSP group ** *p* ≤ 0,002. (**c**) Most ST sections in NSP showed immunostaining for AR (score 1) while there was absence of staining in most STs in KS. (Scale bar = 200 μm; magnification X40 in red dotted square).

In KS a mean of 31% ± 36 % STs did not present the AR (score 0) and a mean of 69% ± 36% STs showed the presence of ARs (score 1). In NSP group a mean of 100% ± 0% STs presented a score 1, in the SCO group a mean of 40% ± 34% STs presented a score 0 and a mean of 60% ± 34% STs a score 1. The differences between the KS and the NSP groups for score 0 (*p* ≤ 0.01) and score 1 (*p* ≤ 0.01) were statistically significant (Figure 2b,c).

### 2.3. Leydig Cells Maturation and Function 

**Figure 3:**
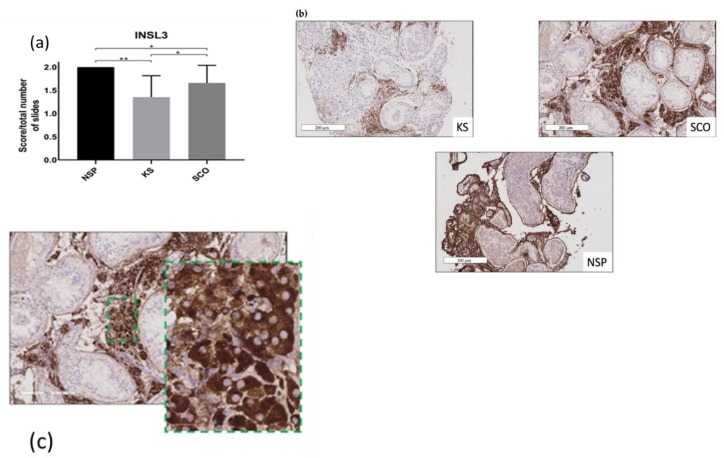
INSL3 expression. Score/total number of slides: (**a**) INLS3 expression is significantly reduced in KS compared to NSP and to SCO * *p* ≤ 0.02 ** *p* ≤ 0.002. (**b**) INSL3 expression in the three populations: KS shows reduction of staining (score 1) compared to NSP and SCO showing almost a complete staining of the interstitium (score 2); (scale bar = 200 μm). (**c**) High magnification of the immunostaining for INSL3 of all cells’ cytoplasm of interstitium in a SCO patient (scale bar = 50 μm in green dotted rectangle).

A total of 256 slides were analysed, with one or two slides (for unilateral or bilateral biopsies) per patient for each protein. A significant reduction of INSL3 expression was observed in KS compared to NSP (*p* ≤ 0.002) and to SCO (*p* ≤ 0.02) (Figure 3a). A mean score of 1.35 ± 0.46, 2 ± 0 and 1.66 ± 0.38 were obtained for the KS, NSP and SCO groups, respectively. 

StAR was expressed in all groups, showing a mean score of 1.41 ± 0.49 in KS, 2 ± 0 for the NSP and 1.45 ± 0.5 for the SCO group with no significant differences found between the three groups of patients.

## 3. Discussion

An important loss of spermatogonia occurs during puberty in KS patients, potentially justifying a prepubertal fertility preservation strategy using cryopreservation of spermatogonial stem cells (SSCs), although this is still highly debated [25]. Indeed, the capacity of KS SSCs to differentiate is not known [26,27]. Two hypotheses that could explain the SSCs’ inability to enter the differentiation process in their original XXY somatic microenvironment have been raised but have not yet been addressed. Considering an increased sperm sex chromosomal aneuploidy in KS [26], either 47,XXY spermatogonia have the potential to complete meiosis explaining both the presence of normal and aneuploid spermatozoa, or spermatozoa only arise from patches of 46,XY SSCs consecutive to “correcting mitotic errors” in the prenatal testis that might give rise to isolated testicular mosaicism, and the increased aneuploid sperm is due to meiotic errors caused by a compromised testicular environment [27,28]. It is therefore important to identify both the molecular mechanisms leading to germ cell loss and cell impairments that may lead to tissue degeneration. We hypothesised that defects from the somatic compartment are involved in seminiferous tubule degeneration.

In the present study we therefore investigated, for the first time in a series of 35 adult KS patients, the expression pattern of several BTB proteins (CNX43, CLD11, ZO1) and of other components of the testicular somatic compartment, comparing them to patients with normal karyotype and either histological testicular tissue degeneration similar to KS (SCO) or normal spermatogenesis (NSP).

In animal studies, it was shown that the BTB is essential for spermatogenesis as CNX43 knockout mice are infertile with small testis and present spermatogenesis arrest at the stage of spermatogonia and STs containing only Sertoli cells. Moreover, CLD11 selective gene knockout murine testes resulted in spermatogenesis arrest [29]. While in men with normal spermatogenesis CLD11 is localised as a linear pattern at the basal compartment of STs, it was shown that in men with non-obstructive azoospermia the protein shifted to the SCs cytoplasm [30,31,32], which suggests that an abnormal expression pattern may also play an important role in human spermatogenesis. Using immunohistochemistry, we demonstrated that in adult KS the tight junction protein CLD11 and the gap junction protein CNX43 were less expressed compared to a population with normal spermatogenesis and SCO patients. Moreover, when present, these proteins presented a less-organised pattern. Surprisingly the percentage of STs with a non-organised pattern (score 1) for CNX43 and CLD11 appeared higher than expected in NSP and higher than in some studies. Indeed, Haverfield et al. [31] analysed three samples with normal spermatogenesis from volunteers for a contraceptive study (not treated) who underwent a testicular needle aspiration and, using four immunoexpression patterns, they observed a high proportion of ‘pattern 2’ (which is the closest to our ‘score 1’) although the proportion was not as high as ours. The difference could also be attributed to a different patient selection for NSP as we used tissue from post-vasectomy patients where over time some modifications within the seminiferous tubules may occur [33], including an increased intraluminal pressure with potential disruption of the BTB that may explain reported anti-sperm antibodies [34,35]. Moreover, others showed a high variability in staining patterns for CLD11 among controls, but they unfortunately did not record the proportion of tubules with the different staining patterns [36].

CLD11 and CNX43 are tethered to the cytoskeleton by adaptor proteins such as ZO proteins that control tight junction structure and function [37]. We studied the ZO1 protein, to ensure that the results we observed were not linked to an absence or a modification of the adapter proteins such as ZO1 connecting tight and gap junctions to the SCs membrane. In our series, in patients with normal spermatogenesis the protein ZO1 was present in a linear fashion at the basolateral location of adjacent SCs. In KS the same distribution pattern of ZO1 was found and no significant difference between the three groups was observed. Hence, we can reasonably assume that the lack of the two proteins CLD11 and CNX43 in KS is not related to a defect of the adaptor protein ZO1.

Besides the BTB proteins, SCs’ function and maturation also have a central role in spermatogenesis and GCs renewal. Indeed, each SC supports a defined number of germ cells [38] and through the secretion of GDNF SCs guarantee SSCs’ renewal [39]. In adult KS patients we found that SCs continued to produce GDNF and no difference was identified in its expression between KS and both NSP and SCO groups. 

With regard to markers of SC maturation, we examined the expression of AMH which is normally produced in prepubertal but not adult SCs [40]. While Wikström et al. in his series of 14 KS boys (aged 10–14 y) showed a postponement of the decrease in serum AMH compared to controls [41], in our series of adult KS, no differences were found when we compared the testis expression of AMH between the three groups and AMH appeared to be absent in most of KS adult patients suggesting that SCs had at least acquired some features of maturation.

Another sign of acquisition of maturity in SCs is the presence of AR. An altered immunoexpression of AR with a reduction of AR nuclear immunostaining was described in prepubertal KS boys compared to age-matched controls [12]. In our series of adult KS we also observed a decreased expression of the nuclear AR compared to the NSP group, but its expression remained similarly low in SCO and KS patients. Therefore, and although testosterone signaling through AR is necessary for the BTB function [13] with previous evidence for a correlation between AR expression and BTB formation [14], we may reasonably assume that disruption in BTB components plays a significant role in the pathophysiology of KS and that the androgen signaling pathway could be involved to a lesser extent. 

Furthermore, a heterogenous profile of SC maturation was observed in a series of seven non-mosaic KS adolescents (aged 15–17 y) when Rives et al. calculated a score for SCs maturation including AMH, AR and vimentin immunoexpression. In their study the authors also suggested that SCs’ maturity was correlated to the persistence of germ cells as in KS patients presenting STs with only Sertoli cells, an immature phenotype was preferentially detected [7]. We did not correlate observations in the somatic compartment with the presence of germ cells. However, in our study there was no difference in AMH expression in KS patients and a reduced AR expression was observed in KS compared to NSP. We may therefore hypothesise that age and patient-specific factors could be responsible for heterogeneous SC maturation markers and BTB protein expression patterns in KS.

We also analysed INSL3, an insulin-like factor secreted by LCs [42] considered as a marker of LC maturity [43]. An interesting observation of our study is the significant reduction of INSL3 in adult KS patients compared to the NSP group but also to the SCO group. This result is in agreement with preliminary observations of Lottrup et al. [43] in five adult KS patients. While we demonstrated features of LCs’ immaturity in KS, we found that the mitochondrial protein StAR was well-expressed in all LCs in our series of KS patients, suggesting that LCs continue to express some proteins required for testosterone biosynthesis. 

Despite a normal or decreased blood circulating testosterone level in KS [44], the reduction of AR and the consequent modification of nuclear signaling essential for spermatogenesis, could be linked, as shown in mice model AR-knock out, to maintenance of spermatogonial numbers, BTB integrity, and completion of meiosis [13].

In conclusion, our results highlight a compromised somatic compartment demonstrated by some abnormal SCs features and the LCs’ lack of maturation. These findings are also in agreement with recent transcriptome studies where D’Aurora et al. showed differential expression of several genes encoding for proteins of both the germinal and somatic compartments. In particular they described genes responsible of steroidogenic function, impairment of inflammatory pathways and BTB structure in six KS adult patients compared to patients with obstructive azoospermia [24,45]. 

In conclusion, to the best of our knowledge, this study involved the largest series of KS adults, providing analysis of the testicular somatic compartment, and for the first time detecting underlying defects in CNX43 and CLD11, the main components of the BTB. 

## 4. Materials and Methods 

### 4.1. Source of Human Tissue

This study was approved by the Ethics Review Board of the Catholic University of Louvain (2017/20OCT/492 on the 20th October 2017). Testicular tissue from three groups of adult patients were selected from the surgery records of the andrology department. Testicular fragments were fixed in 4% paraformaldehyde and were alcohol-dehydrated in xylene and embedded in paraffin. All patients underwent a testicular sperm extraction (TESE) or micro-TESE (microsurgical testicular sperm extraction) procedure for infertility between 2007 and 2017. Thirty-five azoospermic non-mosaic KS patients (mean age 32), nine patients presenting with obstructive azoospermia due to a previous vasectomy after failed PESA (Percutaneous Epididymal Sperm Aspiration) who underwent a testicular biopsy showing normal spermatogenesis at histology and having a normal karyotype (mean age 48), and twenty-eight patients with non-obstructive azoospermia with normal karyotype and an anatomopathological diagnosis of Sertoli cell only syndrome (mean age 32) were included. A total of 128 testicular samples were analysed (see Table 1). Clinical history and patients’ characteristics were recorded from medical files. All other known genetic abnormalities were excluded. 

### 4.2. Immunohistochemical Procedures 

Immunohistochemistry was performed to study the BTB with CNX43, CLD11 and ZO1 antibodies. To assess SCs viability and maturation we used GDNF, AMH and AR antibodies, and to study the LCs maturation and function we used INSL3 and StAR antibodies. References of antibodies are presented in Table 2. Ten sections of 5 μm-thickness were prepared for each patient. Sections mounted on Superfrost Plus slides were deparaffinised and rehydrated. Endogenous peroxidase activity was blocked by incubating the sections with 0.3% H_2_O_2_ for 30 min at room temperature (RT). After washing in deionised water, sections were placed in citrate buffer for 60 min at 98 °C before being washed in 0.05 M Tris-buffered saline (TBS) and 0.05% Triton X-100 and incubated at room temperature (RT) with 10% normal goat serum (NGS, Invitrogen, Merelbeke, Belgium) and 1% bovine serum albumin (BSA, Invitrogen, Merelbeke, Belgium) to block non-specific binding sites for 30 min. The primary antibody was added to the sections and incubated overnight at 4 °C in a humidified chamber. Nonspecific antibody binding was blocked by incubation of samples in 10% NGS and 1% BSA for 30 min at RT. The following day, the slides were washed in 0.05 M TBS and 0.05% Triton X-100 three times for 2 min each and the secondary anti-mouse (for AMH, AR and GDNF) or anti-rabbit (for Claudin 11, Connexin 43, ZO1, INSL3 and StAR) peroxidase-labeled antibody (EnVision+ System-Labeled Polymer-HRP; DAKO K4001 or K4003) was added and incubated for 60 min at RT, followed by washing in 0.05 M TBS and 0.05% Triton X-100 three times for 2 min each. The sections were then incubated for 10 min at RT with the chromogen diaminobenzidine (DAB, DAKO K3468) and nuclei were counterstained with Mayer’s hematoxylin after washing in tap water. For positive controls, human mature testicular tissue with normal spermatogenesis was used. The primary antibody was omitted for negative controls. After dehydrations, sections were mounted and scanned with a Leica SCN400 slide scanner (Leica Biosystems, WETZLAP, Germany). 

### 4.3. Immunohistochemical Evaluation 

For ST counts we considered all seminiferous tubules with an intact basement membrane. Double blind analyses of immunostained slides were performed by M.G.G. and M.V. 

### 4.4. Blood–Testis Barrier 

**Figure 4:**
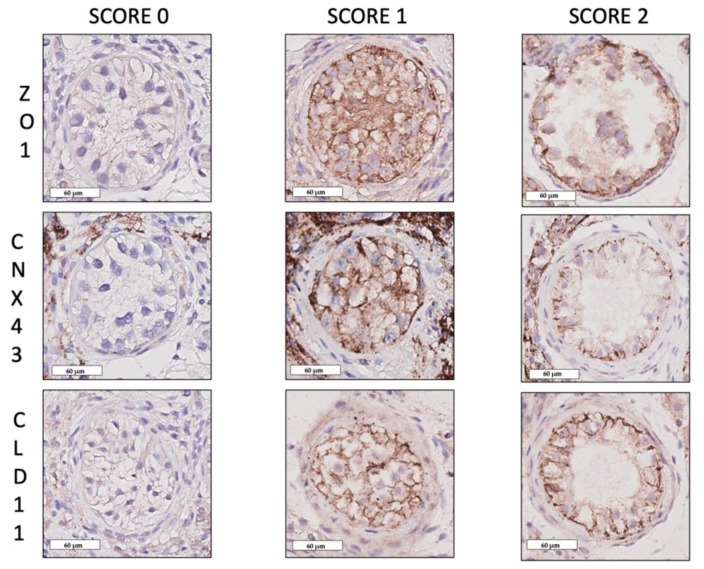
BTB protein scores. Immunostaining scores for zonula occludens-1, connexin-43 and claudin-11. Score 0 was attributed to STs when no staining was observed. Score 1 was attributed to STs when the protein expression appears homogenously diffuse between the apical and the basal compartment. Score 2 was attributed to the normal immunostaining pattern when the protein was localised as a linear pattern on the basal membrane between two SCs (scale bar 60 μm).

We studied a protein for each of the three junction types of the BTB: CLD11, CNX43 and ZO1. A semi-quantitative method (score 0–1–2) was chosen to evaluate the expression of the BTB proteins: CNX43, CLD11, ZO1. A ratio was calculated between numbers of STs of each score and total number of the STs per slide, expressed as a percentage (Figure 1a). A score 0 was attributed to tubules where the protein staining was absent, score 1 to tubules where protein expression was diffuse in the tubules with apical localisation, score 2 to tubules where protein expression was organised only on the basal portion between adjacent SCs (Figure 4). Negative controls were identified by the omission of primary antibody (Figure 1b–d), based on previous experiments on proteins of the BTB where no aspecific staining was observed [14], no neutralisation test was performed besides these negative controls.

### 4.5. Sertoli Cells 

To evaluate the AMH, AR and GDNF immunostainings, we considered the presence (score 1) or the absence of staining (score 0) (Figure 2a–c). The ratio between STs with a given score and the total number of STs was calculated for each slide (Figure 2b). 

### 4.6. Leydig Cells

**Figure 5:**
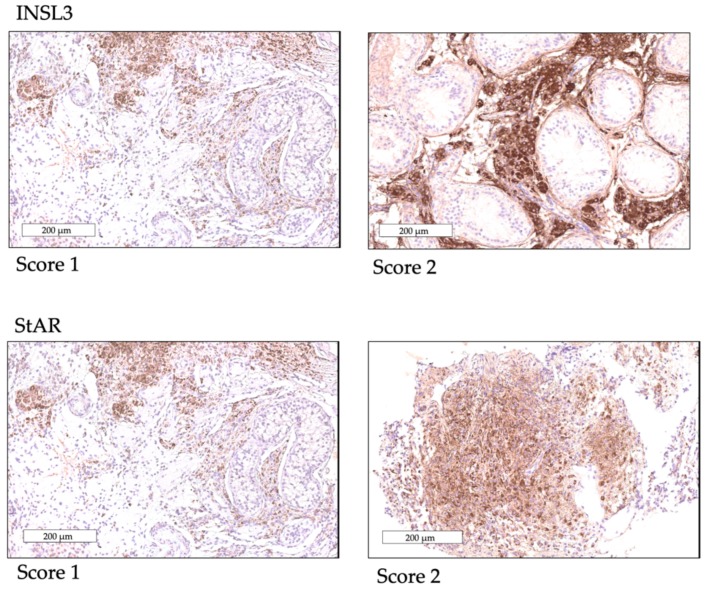
LCs protein expression. Immunostaining scores for INSL3 and StAR. Score 1 partial staining of interstium cells. Score 2 total staining of all interstitium cells. For StAR protein score 2 total staining of a LCs cluster is observed (scale bar 200 μm).

To evaluate LCs, we considered the interstitium staining for INSL3 and StAR (Figure 3b–c, Figure 5) in three fields analysed consecutively from the left corner to the right at 200 μm magnification in order to standardise and homogenise the counting system. We attributed a score to the entire slide: score 2 if there was a staining of the interstitium cells in all the three fields, score 1 if interstium cells were partially marked in one or two of the three fields and score 0 for the absence of staining. A mean of the scores of all 128 slides for each protein was calculated. 

### 4.7. Statistical Analysis

Comparisons between the three groups (KS, SCO and NSP) for each score were tested using a non-parametric Kruskall–Wallis test, followed by Steel–Dwass post-hoc tests with alpha = 0.05 used as the level of significance. 

## 5. Conclusions

In this study we analysed for the first time together three BTB proteins in a big series of 35 adult KS patients. We compared results with NSP and SCO groups to demonstrate that KS adult patients present a reduction of expression of the BTB proteins CLD11 and CNX43 with a different distribution pattern. This pattern was possibly determined by the presence of a dysfunctional somatic compartment with reduced expression of AR in SCs and reduced expression of INSL3 in LCs, both of which are necessary for good cell function and are markers of cell maturity. It remains to be unraveled if KS SSCs are able to differentiate as isolated cells or if another model should be used to replace a compromised somatic compartment necessary for the differentiation process.

## Figures and Tables

**Table 1:** Groups of patients and biopsies numbers analysed.

N Patients	N TESE or μTESE	N Biopsies
35 KS	5 unilateral μTESE 30 bilateral μTESE	65
9 NSP	9 unilateral TESE	9
28 SCO	2 unilateral μTESE 26 bilateral μTESE	54

**Table 2:** Antibodies references and target cells.

Antibody	Target Cell	Dilution	Mono/Polyclonal	Producer	Reference Number
AMH	Sertoli cells	1/200	Monoclonal	Serotec	MICA2246
GDNF	Sertoli cells	1/150	Polyclonal	Sigma-Aldrich	SAB1405863
AR	Sertoli cells	1/100	Monoclonal	DAKO	AR441
CLD11	BTB	1/400	Polyclonal	Santa Cruz	SC25711
CNX43	BTB	1/4000	Polyclonal	Abcam	AB 11370
ZO1	BTB	1/1500	Polyclonal	Sigma-Aldrich	HPA001637
INSL3	Leydig cells	1/2000	Polyclonal	Sigma-Aldrich	HPA028615
StAR	Leydig cells	1/400	Polyclonal	Sigma-Aldrich	HPA027318

## Abbreviations

KS	Klinefelter Syndrome
GCs	Germ Cells
SSCs	Spermatogonial Stem Cells
LCs	Leydig Cells
TESE	Testicular Sperm Extraction
STs	Seminiferous Tubules
SCs	Sertoli Cells
AMH	Anti-Müllerian Hormone
AR	Androgen Receptors
BTB	Blood–Testis Barrier
CLD11	Claudin-11
CNX43	Connexin-43
ZO1	Zonula Occludens-1
INSL3	Insulin-Like Peptide 3
LH	Luteinizing Hormone
StAR	Steroidogenic Acute Regulatory Protein
SCO	Sertoli Cell Only
NSP	Normal Spermatogenesis
GDNF	Glial Cell Line Derived Neurotrophic Factor
